# Association of Low-Value Care Exposure With Health Care Experience Ratings Among Patient Panels

**DOI:** 10.1001/jamainternmed.2021.1974

**Published:** 2021-05-28

**Authors:** Prachi Sanghavi, J. Michael McWilliams, Aaron L. Schwartz, Alan M. Zaslavsky

**Affiliations:** 1 Biological Sciences Division, Department of Public Health Sciences, The University of Chicago, Chicago, Illinois; 2 Department of Health Care Policy, Harvard Medical School, Brigham and Women’s Hospital, Boston, Massachusetts; 3 Division of General Internal Medicine, Department of Medical Ethics and Health Policy, Perelman School of Medicine, Philadelphia, Pennsylvania; 4 Department of Health Care Policy, Harvard Medical School, Boston, Massachusetts

## Abstract

**Importance:**

Patient reviews of health care experiences are increasingly used for public reporting and alternative payment models. Critics have argued that this incentivizes physicians to provide more care, including low-value care, undermining efforts to reduce wasteful practices.

**Objective:**

To assess associations between rates of low-value service provision to a primary care professional (PCP) patient panel and patients’ ratings of their health care experiences.

**Design, Setting, and Participants:**

This quality improvement study used Medicare fee-for-service claims from January 1, 2007, to December 31, 2014, for a random 20% sample of beneficiaries to identify beneficiaries for whom each of 8 low-value services could be ordered but would be considered unnecessary. The study also used health care experience reports from independently sampled beneficiaries who responded to the 2010-2015 Consumer Assessment of Healthcare Providers and Systems (CAHPS) Medicare fee-for-service survey. Statistical analysis was performed from January 1, 2019, to December 9, 2020.

**Main Outcomes and Measures:**

The main outcomes were health care experience ratings from Medicare beneficiaries who responded to the CAHPS survey from 2 domains, namely “Your Health Care in the Last 6 Months” (overall health care, office wait time, timely access to nonurgent care, and timely access to urgent care) and “Your Personal Doctor” (overall personal physician and a composite score for interactions with personal physician). Beneficiaries in both samples were attributed to the PCP with whom they had the most spending. For each PCP, a composite score of low-value service exposure was constructed using the 20% sample; this score represented the adjusted relative propensity of the PCP patient panel to receive low-value care. The association between low-value service exposure and health care experience ratings reported by the CAHPS respondents in the PCP patient panel was estimated using regression analysis.

**Results:**

The final sample had 100 743 PCPs, with a mean of approximately 258 patients per PCP. Only 1 notable association was found; more low-value care exposure was associated with more frequent reports of having to wait more than 15 minutes after the scheduled time of an appointment (a mean of 0.448 points lower CAHPS score on a 10-point scale for PCP patient panels who received the most low-value care vs the least low-value care). Although some other associations were statistically significant, their magnitudes were substantially smaller than those typically considered meaningful in other CAHPS literature and were inconsistent in direction across levels of low-value service exposure.

**Conclusions and Relevance:**

This quality improvement study found that more low-value care exposure for a PCP patient panel was not associated with more favorable patient ratings of their health care experiences.

## Key Points

**Question:**

Do primary care professional (PCP) patient panels who receive more low-value care rate their health care experiences more favorably?

**Findings:**

This quality improvement study of 100 743 PCPs with a mean of 258 patients each constructed a composite score of low-value care exposure for each PCP patient panel and estimated its association with patient ratings of health care. With 1 exception (waiting room time), all observed associations between low-value care exposure and health care experience ratings (overall health care, timely access to nonurgent care, timely access to urgent care, personal physician, and interactions with personal physician) were small and/or lacked statistical significance.

**Meaning:**

This study did not find evidence that more low-value care for a PCP patient panel was associated with more favorable patient ratings of their health care experiences.

## Teaser

This quality improvement study assesses associations between rates of low-value service provision to a primary care professional patient panel and patients’ ratings of their health care experiences.

## Introduction

Patient-reported health care experiences are widely used to incentivize quality improvement through public reporting and performance-based payments. For example, the Consumer Assessment of Healthcare Providers and Systems (CAHPS) survey measures of patient experiences are included in Medicare Advantage plan star ratings^1^ and the Medicare Shared Savings Program accountable care organization contracts.^2^ These ratings measure dimensions of care that are best reported by patients, such as physician communication and timeliness. They are empirically associated with other measures of clinical process, outcomes, efficiency, and safety.^3,4,5^ A concern about this practice is that it may encourage physicians to provide more low-value services (care that is not associated with a clinical benefit), out of a belief that responding to patient demand or the perception that more care is better will improve their ratings.^6,7,8,9^ This may lead to the wasteful use of health care resources and spending, possible iatrogenic injury, and limited success of alternate payment models such as accountable care organizations.^10^

In this study, we directly address the question: Do patients of physicians who provide more low-value care rate their health care experiences more favorably? (We use the terms *receipt of*, *exposure to*, or *provision of* low-value care interchangeably, regardless of how or by whom it was initiated.) More low-value care may be interpreted by patients as a signal of better or worse care depending on patient trust and preferences, as well as physician communication of rationales.^3,11,12,13^ It remains unclear whether patients are advocating for low-value service provision.^10,11,14,15,16,17^ However, as long as physicians may be acting out of a concern about patient dissatisfaction, it is imperative to address this question to gain physician buy-in to policies for curbing waste.

One study that garnered national attention found that patients who reported most favorably on their care and physician communication had a higher mortality risk, implying that catering to patient satisfaction may lead to worse outcomes.^18^ However, the additional attention that patients with severe illness receive may lead them to rate their access and communication with physicians more favorably than would healthier patients with fewer care needs. This scenario may induce a positive correlation between patient experiences and mortality that is actually a reflection of patient factors, not the associations of physician practices with patient experiences. The limitation of the study design that made it vulnerable to this potentially flawed inference is its reliance on data from the same unique patient for both the patient report and the outcome.^3,4,6,19^

In the context of our research question, similar confounding due to patient characteristics can arise if such factors are associated with both receipt of low-value care and health care experiences. For example, some patients may have more opportunities to receive low-value care than other patients in similar states of health because they value health care more and thus seek care more frequently. Likewise, patients who comply with physician orders more often are likely to receive more services. Patients with such stronger preferences or adherence may also appreciate their care and physicians more. Consequently, receipt of low-value care may be associated with more favorable reports on care experiences from the same patients even when physicians’ low-value practice patterns are not associated with their patients’ care experiences, and even when patients do not differ between physicians.

We designed a study that eliminates this source of patient-level confounding. We used a 20% random sample of the full fee-for-service Medicare population to assess low-value care exposure for a primary care professional (PCP) patient panel and a much smaller, independent sample from the CAHPS survey to measure patient experiences. Because the 2 samples were independent and overlapped minimally, our analysis did not rely on observations about the same patients to assess associations. Although our study remains subject to unmeasured confounding arising from unobserved systematic sorting of patients to different PCPs, our methods permit stronger conclusions about the associations between physician provision of wasteful care and care experiences than approaches taken in prior studies.

## Methods

### Data Sources

To assess provision of low-value services, we analyzed claims and enrollment data from January 1, 2007, to December 31, 2014, for a 20% simple random sample of fee-for-service Medicare beneficiaries, with the first year serving as a look-back period for 2008 claims. We used hospital inpatient data, outpatient claims, and noninstitutional claims for services such as physician visits. For a given year, we required beneficiaries to be continuously enrolled in Medicare Parts A and B in that year (while alive) and the previous year. Eligible beneficiaries also had at least 1 claim for primary care services (Healthcare Common Procedure Coding System codes 99201-99215, G0402, G0438, and G0439) provided by a PCP, defined by specialty codes for general practice, family practice, internal medicine, or geriatric medicine (eAppendix 1 in the Supplement); we attributed beneficiaries to the PCP with whom they had the most spending during the year.^20^ The research protocol was approved by institutional review boards at Harvard Medical School, the University of Chicago, and the National Bureau of Economic Research with a waiver of informed consent according to CFR §46.116 (e)(3)(ii) because the research could not practicably be carried out without the waiver.

We assessed patient experiences with data from the 2010-2015 fee-for-service Medicare CAHPS surveys. We assigned each CAHPS respondent to a PCP using the approach described but linked to a PCP in the prior year because the CAHPS survey is administered near the beginning of the year and asks about the prior 6 months. The overall CAHPS survey response rate was 41.9% during these years. The nonresponse rates to items in the CAHPS ranged from 3% to 67%, almost entirely owing to inapplicability of the item to a respondent who did not have relevant experience (eAppendix 2 and eTable 1 in the Supplement). Because the CAHPS sample was only 1.5% the size of the claims sample used to assess low-value service provision, the overlap between the 2 samples was minimal.

We excluded PCPs with fewer than 11 patients in the 20% sample (in compliance with our data use agreement with the Centers for Medicare & Medicaid Services) or without at least 1 attributed CAHPS respondent (eAppendix 3 and eFigure in the Supplement). Clinical covariates were extracted from the Chronic Conditions Data Warehouse,^21^ which tracks diagnoses of 27 conditions from 1999 or a beneficiary’s first year of Medicare enrollment onward, zip code–level sociodemographic data from the American Community Survey in the same years as our claims data, and data on hospital referral regions from the Dartmouth Atlas.^22^

### Study Variables

#### Low-Value Service Exposure for PCP Patient Panels

To create measures of exposure to low-value care for each PCP-attributed patient panel in the 20% samples, we started by identifying episodes of low-value care at the patient level. We adapted methods developed by Schwartz et al^16,23^ for identifying low-value care in Medicare claims data and selected the 8 services that were most frequently used (Table 1). In addition, for each service, we defined and identified a denominator population among whom the service would be considered unnecessary (eAppendix 4 and eTable 2 in the Supplement). Although the services are often ordered by PCPs, our approach did not require that the PCP provided or ordered the low-value service; rather, we assessed low-value service exposure from all physicians of a PCP patient panel. The contribution of a PCP’s network of specialists to the provision of low-value care was compatible with several dimensions of care experiences examined in our analysis. For example, overall care ratings are not PCP specific, and overall PCP ratings may reflect patients’ valuation of specialty referrals made by their PCP.

**Table 1.** Definitions and Frequencies of Low-Value Services in the Medicare Fee-for-Service Population

Low-value service description	Denominator population for which service might be considered low value	Specific procedure and scenario criteria for identifying low-value service receipt	No. (%)		
			Population in denominator^a^	Denominator that received service^b^	Population that received service^c^
**Screenings and tests**						
PSA testing in older male patients	Male patients aged ≥75 y with no history of prostate cancer	PSA test	5 002 928 (13.5)	2 101 823 (42.0)	2 101 823 (5.7)
Screening for carotid artery disease in asymptomatic adults	Patients with no history of stroke or TIA prior to index year	Carotid imaging not associated with inpatient or emergency care without a diagnosis of stroke, TIA, or focal neurologic symptoms on claim	32 257 582 (86.8)	1 950 920 (6.1)	1 950 920 (5.3)
Cervical cancer screening for older female patients	Female patients aged ≥65 y with no cervical cancer, dysplasia, diagnoses of other female genital cancers, abnormal Papanicolaou test findings, or human papillomavirus positivity noted in index year’s claims or in prior year’s claims	Screening Papanicolaou test	17 939 421 (48.3)	1 456 682 (8.1)	1 456 682 (3.9)
Parathyroid hormone test for patients with stage 1-3 CKD	Patients with CKD, with no hypercalcemia diagnosis noted in index year’s claims	PTH test with no dialysis service within 30 d after test	7 765 654 (20.9)	795 137 (10.2)	795 137 (2.1)
Total or free T3 level testing for patients with hypothyroidism	Patients with hypothyroidism diagnosis in index year’s claims	Total or free T3 measurement	4 394 744 (11.8)	638 415 (14.5)	638 415 (1.7)
**Imaging and treatment**						
Back imaging for nonspecific low back pain	Patients with no diagnoses for cancer, trauma, intravenous drug abuse, neurologic impairment, endocarditis, septicemia, tuberculosis, osteomyelitis, fever, weight loss, loss of appetite, night sweats, anemia, radiculitis and myelopathy, and no back imaging after 6 wk of first diagnosis of low back pain, in index year’s claims	Back imaging with a diagnosis of low back pain within 6 wk of first diagnosis of low back pain	36 953 430 (99.4)	1 499 313 (4.1)	1 499 313 (4.0)
Head imaging for uncomplicated headache	Patients with no diagnoses for thunderclap headache, epilepsy, giant cell arteritis, head trauma, convulsions, altered mental status, nervous system symptoms (eg, hemiplegia), disturbances of skin sensation, speech problems, stroke or TIA, history of stroke, or cancer in index year’s claims	Brain CT scan or MRI	36 717 814 (98.8)	913 839 (2.5)	913 839 (2.5)
Spinal injection for low back pain	Patients with no diagnoses for radiculopathy in index year’s claims, and no patients with spinal injections within 14 d after an inpatient stay	Outpatient epidural (not indwelling), facet, or trigger point injections with diagnosis for low back pain	36 379 131 (97.9)	599 114 (1.7)	599 114 (1.6)

Abbreviations: CKD, chronic kidney disease; CT, computed tomography; MRI, magnetic resonance imaging; PSA, prostate-specific antigen; PTH, parathyroid hormone test; T3, triiodothyronine; TIA, transient ischemic attack.

^a^ Percentage of beneficiaries who met the denominator criteria in column 2 among fee-for-service Medicare beneficiaries from 2008 to 2014.

^b^ Percentage of beneficiaries who received at least 1 provision of the low-value service among those in the denominator for that service.

^c^ Percentage of beneficiaries who received at least 1 provision of low-value service among fee-for-service Medicare beneficiaries from 2008 to 2014. This is also the product of columns 4 and 5.

We aggregated the beneficiary-level indicators of low-value service receipt into composite measures of exposure at the level of a PCP-attributed patient panel using a multivariate, multilevel model with the patients nested within the PCPs: logit Pr (LVS_Receipt*_ijst_* = 1) = *α_j_* *+* *X_ijst_*′β*_s_* and *α_j_* ~ *N*(0, σ^2^), where LVS_Receipt*_ijst_* is the binary indicator of whether patient *i* of PCP *j* received low-value service *s* in year *t*, and *X_ijst_* is a vector of patient- and geography-related covariates (zip code–level covariates and hospital referral region) and year indicators. Exposure to low-value care for the patient panel of PCP *j* is represented by the random intercept *α_j_*, which represents the relative propensity to receipt of low-value services among the patients in the PCP patient panel, after controlling for the vector of covariates in *X_ijst_*, and accounts for sampling variation in the number of patients and potential services per PCP (eAppendix 5 in the Supplement).

#### CAHPS Measures

We focused on 9 items in 2 sections of the CAHPS Medicare fee-for-service survey: “Your Health Care in the Last 6 Months” and “Your Personal Doctor,” defined as the “one you would see if you need a checkup, want advice about a health problem, or get sick or hurt” (Table 2). In the former section, we studied responses to questions about overall health care, waiting room times, and timely access to nonurgent and urgent care. In the latter, we assessed ratings of the personal physician overall and interactions with the personal physician. All of these items are commonly studied in the CAHPS literature^24,25^ and could conceivably be associated with provision of low-value care.

**Table 2.** Medicare Fee-for-Service CAHPS Survey Items of Patients' Experiences With Care

Survey item^a^	Survey question	Original scale^b^
**Your health care in the last 6 mo**		
Overall rating of health care	What number would you use to rate all your health care in the past 6 mo?	0-10
Appointment waiting time	In the last 6 mo, how often did you see the person you came to see within 15 min of your appointment time?	1-4
Timely access to nonurgent care	In the last 6 mo, how often did you get an appointment for a checkup or routine care as soon as you needed?	1-4
Timely access to urgent care	In the past 6 mo, when you needed care right away, how often did you get care as soon as you thought you needed it?	1-4
**Your personal doctor**		
Overall rating of personal physician	What number would you use to rate your personal doctor?	0-10
**Interactions with personal doctor (composite subquestions)**^c^		
Clear communication	In the past 6 mo, how often did your personal doctor explain things in a way that was easy to understand?	1-4
Careful listening	In the past 6 mo, how often did your personal doctor listen carefully to you?	1-4
Respect	In the past 6 mo, how often did your personal doctor show respect for what you had to say?	1-4
Sufficient time	In the past 6 mo, how often did your personal doctor spend enough time with you?	1-4

Abbreviation: CAHPS, Consumer Assessment of Healthcare Providers and Systems

^a^ All items existed in the 2010-2015 surveys used for this analysis.

^b^ All responses were rescaled to a 10-point scale for analysis.

^c^ Beneficiary-level composites scores were created for these subquestions by averaging across standardized responses.

All responses were linearly rescaled to a 0 to 10 scale for consistency in presentation of results (Table 2). We created patient-level composite scores for respondents’ interactions with their personal physician by averaging across standardized responses (ie, by first subtracting the grand item mean from individual responses), averaging across de-meaned responses, and adding back the mean of all grand item means. We did not expect bias from different nonresponse among these items because 98.8% of patients who responded to at least 1 of these items responded to all of the items.

#### Covariates

Patient covariates in the model estimating low-value service exposure included age, sex, race/ethnicity, Medicaid-Medicare dual status in at least 1 month, and indicators for 27 chronic conditions. To further adjust for health status, we added an indicator of having 6 or more chronic conditions and also used the claims data to calculate Hierarchical Condition Category risk scores, which indicate higher future Medicare spending with higher scores. Zip code–level sociodemographic variables included median household income, percentage of patients in poverty, and percentage of patients with a college education.

For the main analysis of an association between CAHPS scores and low-value service exposure, we included the following patient variables from the CAHPS survey: age as a categorical variable (<65, 66-69, 70-74, 75-79, 80-84, and ≥85 years), Medicaid-Medicare dual eligibility status, highest level of education completed (less than high school, some high school, some college, college graduate, and above college), and self-reported overall physical health and overall mental or emotional health (poor, fair, good, very good, or excellent).

### Statistical Analysis

Statistical analysis was performed from January 1, 2019, to December 9, 2020. We categorized PCPs into deciles of the low-value service composite for their attributed patients, from least (decile 1) to most (decile 10) low-value care exposure. We first regressed CAHPS scores on decile indicators and patient covariates, with clustered standard errors to account for patient grouping within physicians, for a 9-*df* omnibus test of any differences across deciles. Our second analysis regressed CAHPS scores on the numerical decile index and covariates to test for a linear trend across deciles. To further describe trends, for each CAHPS model, we conducted statistical tests of the difference between the mean adjusted CAHPS score in each decile of the low-value service exposure composite and the overall adjusted mean CAHPS score. All statistical tests were 2-sided and conducted at an α level of .05.

## Results

Approximately 26 million beneficiary-years were used to create the low-value service composites for 100 743 PCP patient panels, with a mean of approximately 258 patients per PCP. Depending on the CAHPS item, there were between 135 657 and 330 600 respondents, ranging from 2 to 3.4 per PCP.

In outpatient facility claims for low-value services, the PCP was listed on 41.8% of services, as the attending physician (31.4%), operating physician (2.9%), or other physician (7.5%). Among professional claims for low-value services, the PCP was listed on 45.6% of services, either as the performing physician (10.0%) or referring physician (35.6%).

**Figure 1.**
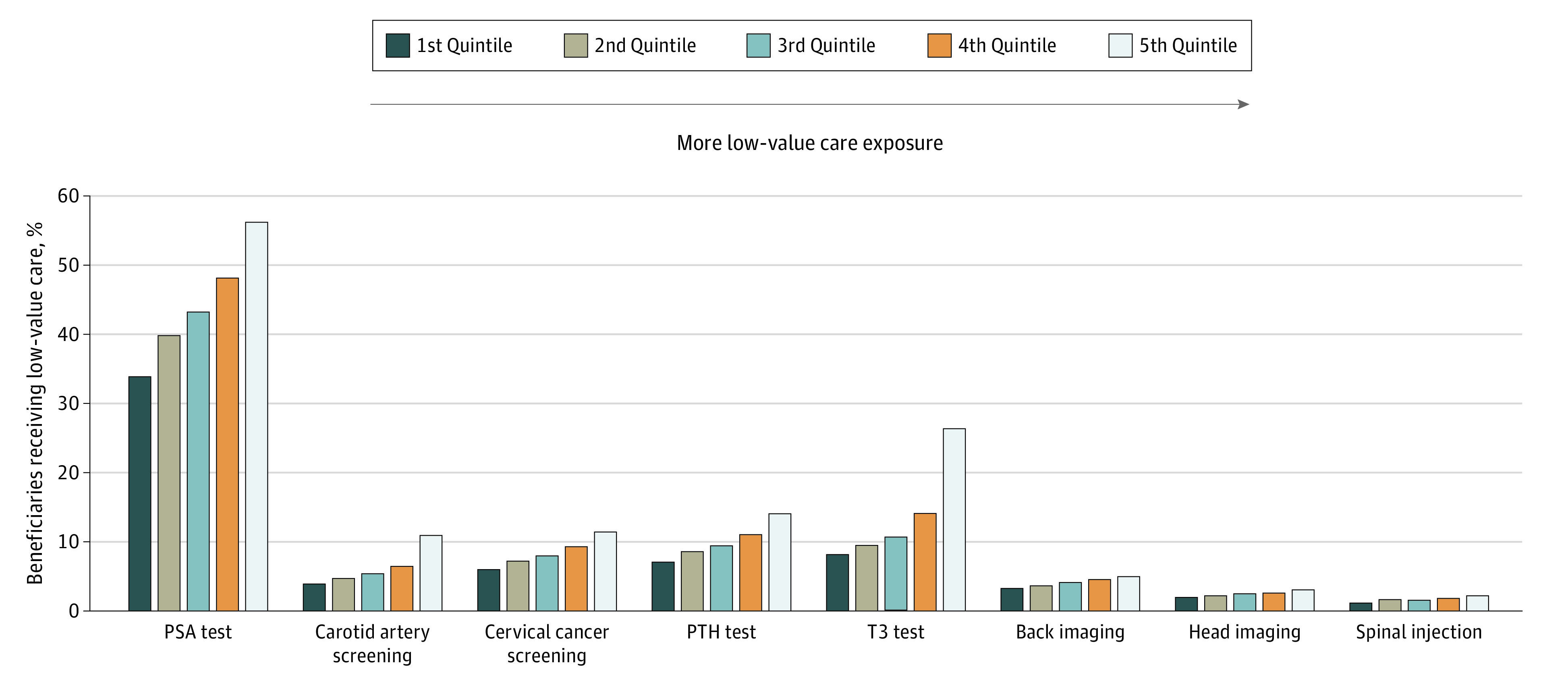
Mean Percentage of Beneficiaries Receiving Specific Low-Value Services by Quintile of Low-Value Service Exposure Mean percentage of low-value service receipt is calculated by first computing, for each primary care professional (PCP) patient panel, the percentage of beneficiaries who received the service among those in the denominator population for that service, and then averaging those percentages across PCP patient panels. The first quintile represents the PCP patient panels with the least low-value care exposure and the fifth quintile represents the PCP patient panels with the most low-value care exposure. PSA indicates prostate-specific antigen; PTH, parathyroid hormone; and T3, triiodothyronine.

We observed substantial variation in low-value care exposure across patient panels, consistent with prior studies.^20^ Figure 1 shows that the low-value service exposure composites are positively associated with use in each of the 8 services. We did not find meaningful differences between patient panels in the first and fifth quintiles of low-value service exposure (eAppendix 6 and eTables 3 and 4 in the Supplement).

**Figure 2.**
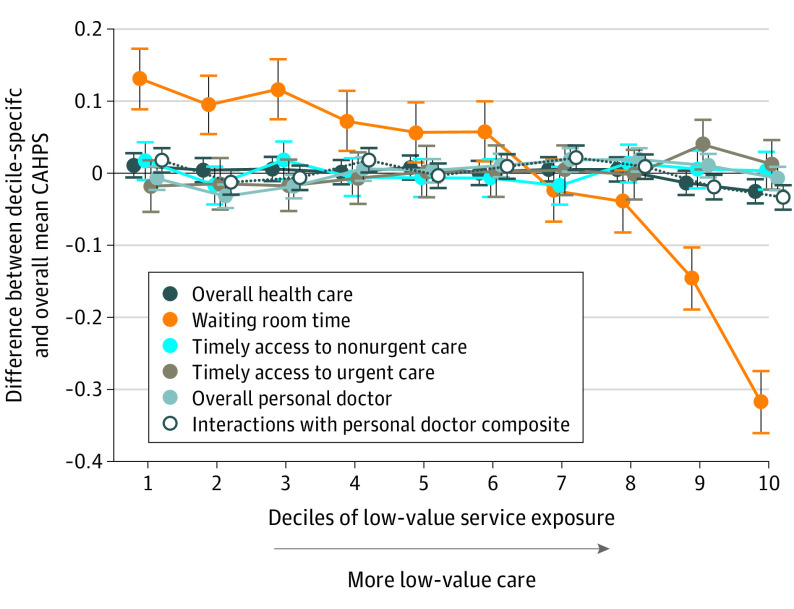
Differences Between Consumer Assessment of Healthcare Providers and Systems (CAHPS) Scores at Levels of Low-Value Service Exposure and Overall Mean CAHPS Score Each CAHPS outcome was separately modeled with a linear regression that adjusted for age, Medicaid-Medicare dual status, highest level of education completed, overall physical health rating, and overall mental or emotional health rating and included physician-clustered SEs. Trend lines are horizontally offset by small amounts (0.8 deciles) for readability. Vertical bars indicate 95% CIs.

For each CAHPS outcome, Figure 2 plots differences between the mean adjusted CAHPS score for each decile of the low-value service exposure measure and the overall mean adjusted CAHPS score across deciles. With the exception of waiting room time, trend coefficients were bounded to be very close to zero. The waiting room time item had a clear downward trend, with lower CAHPS scores (reporting more frequently late start of appointments) in the deciles of patients receiving the most low-value care. In particular, PCP patient panels who received the most low-value care responded with a CAHPS score that was a mean of 0.448 points lower than those who received the least low-value care, a difference 7.7 times larger than the maximum difference observed between any 2 deciles in any other outcome.

Table 3 summarizes the mean adjusted CAHPS scores across deciles of the low-value service exposure measure. With the exception of waiting room time, mean adjusted scores in each decile were above 8.5, with a maximum between-decile difference of 0.058, on a 10-point scale. A test of linear trend across the deciles found significant trends of CAHPS scores for 5 of 6 measures (compared with only 3 for the categorical specification). However, excluding waiting room time, the largest-magnitude coefficient indicated that a 1 higher decile of low-value service exposure would increase the CAHPS outcome by a mean of only 0.005 points, adjusting for other covariates. We also provide full regression tables (eAppendix 7 and eTables 5-8 in the Supplement), sensitivity analyses (eAppendices 8 and 9 and eTables 9 and 10 in the Supplement), and description of all code files (eAppendix 10 and eTables 11-15 in the Supplement).

**Table 3.** Mean Adjusted CAHPS Scores by Low-Value Service Exposure^a^

Low-value service exposure	Your health care in the last 6 months				Your personal doctor	
	Overall health care	Waiting time	Timely access to nonurgent care	Timely access to urgent care	Overall personal doctor	Interactions with personal doctor composite
Deciles of low-value service exposure, specified as categorical variable^b^						
1	9.160	6.540	8.594	8.933	9.471	9.545
2	9.153	6.504	8.560	8.936	9.446	9.513
3	9.155	6.526	8.596	8.934	9.459	9.522
4	9.152	6.482	8.572	8.943	9.482	9.545
5	9.157	6.466	8.571	8.953	9.481	9.523
6	9.150	6.467	8.570	8.953	9.489	9.535
7	9.155	6.384	8.559	8.956	9.495	9.547
8	9.155	6.371	8.590	8.948	9.497	9.537
9	9.135	6.263	8.582	8.991	9.488	9.507
10	9.125	6.092	8.581	8.963	9.470	9.493
*F* statistic^b^	1.51	37.21	0.88	0.91	3.56	3.55
*P* value	.14	<.001	.54	.51	<.001	<.001
Deciles of low-value service exposure, specified as continuous variable^c^						
Low-value service exposure	−0.003	−0.041	−0.0002	0.005	0.003	−0.003
*P* value	.006	<.001	.92	.02	.001	.02

Abbreviation: CAHPS, Consumer Assessment of Healthcare Providers and Systems.

^a^ Each CAHPS outcome was separately modeled with a linear regression that adjusted for age, Medicaid-Medicare dual status, highest level of education completed, overall health rating, and overall mental or emotional health rating, and included physician-clustered SEs.

^b^ Deciles of low-value service exposure were specified as categorical variables and the intercept was dropped to allow direct interpretation of decile coefficients as mean adjusted CAHPS scores (rather than as comparisons with a reference category). *F* tests were conducted to test the joint significance of the decile coefficients. As an example of interpretation, primary care professional patient panels in the fifth decile of low-value care exposure rated their overall health care 9.157 out of 10, on average, controlling for age, dual status, educational level, and overall health and mental or emotional health rating.

^c^ Deciles of low-value service exposure were specified as a continuous variable (integers 1-10). As an example of interpretation, primary care professional patient panels in one higher decile of low-value care exposure rated their overall health care 0.003 points lower (on a 10-point scale), on average, controlling for age, dual status, educational level, and overall health and mental or emotional health rating.

## Discussion

We assessed the associations between PCP patient panels’ levels of exposure to low-value care and health care experience ratings. Across multiple items from CAHPS sections on “Your Health Care in the Last 6 Months” and “Your Personal Doctor,” we did not find that exposure to more low-value care was associated with better health care experience ratings. Although some associations were statistically significant, their magnitudes were substantially smaller than those typically considered meaningful in other CAHPS literature^26,27^ and inconsistent in direction across levels of low-value service exposure. These findings challenge the claim that use of patient-reported experiences to measure quality of care may lead to unnecessary care and possibly iatrogenic injury and death.^6,7,8,9^

The only notable association was that patient panels with higher exposure to low-value care reported more frequently having behind-schedule appointments. Specifically, patient panels with the highest low-value care exposure rated this item 0.448 points lower on a 10-point scale than those with the lowest low-value care exposure, a difference substantially larger than typically observed in comparisons of Medicare plans.^26^ One possible interpretation is that poorly organized or overwhelmed practices substitute wasteful services for higher-value services that require more cognitive effort and clinician time.^28,29^

Our study design, however, was correlational and did not support conclusions about causal mechanisms underlying any associations between low-value service provision and patient experience reports. It improves on the prior literature, which has been characterized by patient-level endogeneity from using the same individuals to measure both patient experiences and health outcomes or health care use.^7,18^ For the research question our study addressed, such an analysis may potentially misattribute to receipt of low-value care what is actually an association of unobserved patient characteristics, such as preferences for amount and type of care; socioeconomic factors, such as educational level, income, and leisure time; and trust in their physician and the health care system more broadly. Our study design reduced this type of patient-level confounding by using independent samples, instead of the same sample, to assess low-value care provision and health care experiences. Furthermore, because the CAHPS sample was much smaller than the claims sample, there was little overlap between the 2 samples. In other words, patient-level confounding was necessarily limited as the characteristics of a CAHPS respondent were at most weakly associated with the mean characteristics of the PCP patient panel. The large claims sample also made possible a more precise estimation of low-value care exposure, and the variation in the estimated exposure supported informative comparisons of health care experiences between higher and lower levels of low-value care provision.

### Limitations

This study has limitations. First, we relied on claims data to identify diagnoses and procedures, which may not be as complete or accurate as medical records. Second, our analysis does not address systematic, unobserved differences in patient panels between physicians. For example, patients may sort to physicians based on the consonance between their preferences and a physician’s practice style. Third, although the CAHPS response rate was 41.9%, our interest was in the reports of patients who respond to such surveys. This response rate is typical of surveys of this type, and the respondents are likely representative of those who would respond to other patient surveys.

Fourth, to measure low-value service exposure, we counted all low-value services received by patients in a PCP patient panel, thereby attributing the provision of low-value care to the network of physicians who provided care to the PCP patient panel. Such PCP-centric models for identifying and establishing care networks are commonly used by researchers and policy makers.^30,31,32,33,34,35^ Because only a share of low-value services were provided by a patient’s PCP, associations between low-value care provision and PCP-specific patient ratings may have been attenuated toward the null. However, we found similarly weak associations for CAHPS survey items associated with health care more broadly, including patients’ overall care rating, which may reflect care provided by all physicians involved in the patient’s care.

## Conclusions

We did not find evidence that patients who are exposed to more low-value care rate their health care experiences better. They also generally do not rate their health care worse, which may be because they are unable to assess the value of care or because their physicians effectively correct information asymmetries through shared decision-making when denying low-value services. These findings are inconsistent with some highly cited works,^7,18^ likely in part because our methodological approach is not subject to the same levels of patient-level confounding. Knowing this may help reduce the use of low-value care that is provided to appease patients who would be equally satisfied with less wasteful care and help alleviate concerns that patient dissatisfaction should inhibit waste reduction under alternative payment models.

## Notes

Supplement. eAppendix 1. Identifying Primary Care Providers eAppendix 2. Missingness of CAHPS Items eTable 1. Percent of Missing Responses by CAHPS Item and Year eAppendix 3. Low-Value Service Exposure Analytical Sample Flowchart eFigure. Low-Value Service Exposure Analytical Sample Flowchart eAppendix 4. Comparison of Denominator Descriptions Used by Sanghavi et al vs Schwartz et al eTable 2. Comparison of Denominator Descriptions Used by Sanghavi et al vs Schwartz et al eAppendix 5. Low-Value Service Exposure Modeling Details eAppendix 6. Average Characteristics of PCP Patient Panels in First and Fifth Low-Value Service Exposure Composite Quintiles eTable 3. Average Characteristics of PCP Patient Panels in First and Fifth Low-Value Service Exposure Composite Quintiles, Unweighted eTable 4. Average Characteristics of PCP Patient Panels in First and Fifth Low-Value Service Exposure Composite Quintiles, Weighted by Panel Size eAppendix 7. Full Regression Tables eTable 5. Coefficients and *P* Values for “Fixed Effects” Sub-Step Regression by Low-Value Service eTable 6. Coefficients and *P* Values for Low-Value Service Exposure Composite Model eTable 7. Coefficients, Standard Errors, and *P* Values for CAHPS Linear Regressions for Non-Composite Items eTable 8. Coefficients, Standard Errors, and *P* Values for CAHPS Linear Regressions for Interactions With Personal Doctor Composite eAppendix 8. Additional Analysis: Estimating Low-Value Service Exposure Using Only More Physician-Driven Services eTable 9. Average Adjusted CAHPS Scores by Low-Value Service Exposure, Based on Only More Physician-Driven Services eAppendix 9. Additional Analysis: No Covariate Adjustment in CAHPS Model eTable 10. Average Unadjusted CAHPS Scores by Low-Value Service Exposure eAppendix 10. Description of all Code Files, With Website Address for GitLab Download eTable 11. Files Related With Identifying Low-Value Services eTable 12. Files Related With Identifying Primary Care Providers eTable 13. Files Related With Applying Further Denominator Exclusions eTable 14. Files Related With Creating Sample Summary Statistics and Adding Additional Covariates eTable 15. Files Related With Modeling and Analysis
