# Supplementary material for: Association of Low-Value Care Exposure With Health Care Experience Ratings Among Patient Panels
Source: JAMA Intern Med. 2021 May 28;181(7):1–9. doi: 10.1001/jamainternmed.2021.1974 (PMC8261613; doi:10.1001/jamainternmed.2021.1974)
Supplement: Supplement. — eAppendix 1. Identifying Primary Care Providers eAppendix 2. Missingness of CAHPS Items eTable 1. Percent of Missing Responses by CAHPS Item and Year eAppendix 3. Low-Value Service Exposure Analytical Sample Flowchart eFigure. Low-Value Service Exposure Analytical Sample Flowchart eAppendix 4. Comparison of Denominator Descriptions Used by Sanghavi et al vs Schwartz et al eTable 2. Comparison of Denominator Descriptions Used by Sanghavi et al vs Schwartz et al eAppendix 5. Low-Value Service Exposure Modeling Details eAppendix 6. Average Characteristics of PCP Patient Panels in First and Fifth Low-Value Service Exposure Composite Quintiles eTable 3. Average Characteristics of PCP Patient Panels in First and Fifth Low-Value Service Exposure Composite Quintiles, Unweighted eTable 4. Average Characteristics of PCP Patient Panels in First and Fifth Low-Value Service Exposure Composite Quintiles, Weighted by Panel Size eAppendix 7. Full Regression Tables eTable 5. Coefficients and P Values for “Fixed Effects” Sub-Step Regression by Low-Value Service eTable 6. Coefficients and P Values for Low-Value Service Exposure Composite Model eTable 7. Coefficients, Standard Errors, and P Values for CAHPS Linear Regressions for Non-Composite Items eTable 8. Coefficients, Standard Errors, and P Values for CAHPS Linear Regressions for Interactions With Personal Doctor Composite eAppendix 8. Additional Analysis: Estimating Low-Value Service Exposure Using Only More Physician-Driven Services eTable 9. Average Adjusted CAHPS Scores by Low-Value Service Exposure, Based on Only More Physician-Driven Services eAppendix 9. Additional Analysis: No Covariate Adjustment in CAHPS Model eTable 10. Average Unadjusted CAHPS Scores by Low-Value Service Exposure eAppendix 10. Description of all Code Files, With Website Address for GitLab Download eTable 11. Files Related With Identifying Low-Value Services eTable 12. Files Related With Identifying Primary Care Providers eTable 13. Files Related With A [file jamainternmed-e211974-s001.pdf]

Supplementary Online Content

Sanghavi P, McWilliams JM, Schwartz AL, Zaslavsky AM. Association of low-value care exposure with health care experience ratings among patient panels. *JAMA Intern Med.* Published online May 28, 2021. doi:10.1001/jamainternmed.2021.1974

eAppendix 1. Identifying Primary Care Providers
eAppendix 2. Missingness of CAHPS Items
eTable 1. Percent of Missing Responses by CAHPS Item and Year
eAppendix 3. Low-Value Service Exposure Analytical Sample Flowchart
eFigure. Low-Value Service Exposure Analytical Sample Flowchart
eAppendix 4. Comparison of Denominator Descriptions Used by Sanghavi et al vs Schwartz et al
eTable 2. Comparison of Denominator Descriptions Used by Sanghavi et al vs Schwartz et al
eAppendix 5. Low-Value Service Exposure Modeling Details
eAppendix 6. Average Characteristics of PCP Patient Panels in First and Fifth Low-Value Service Exposure Composite Quintiles
eTable 3. Average Characteristics of PCP Patient Panels in First and Fifth Low-Value Service Exposure Composite Quintiles, Unweighted
eTable 4. Average Characteristics of PCP Patient Panels in First and Fifth Low-Value Service Exposure Composite Quintiles, Weighted by Panel Size
eAppendix 7. Full Regression Tables
eTable 5. Coefficients and *P* Values for “Fixed Effects” Sub-Step Regression by Low-Value Service
eTable 6. Coefficients and *P* Values for Low-Value Service Exposure Composite Model
eTable 7. Coefficients, Standard Errors, and *P* Values for CAHPS Linear Regressions for Non-Composite Items
eTable 8. Coefficients, Standard Errors, and *P* Values for CAHPS Linear Regressions for Interactions With Personal Doctor Composite
eAppendix 8. Additional Analysis: Estimating Low-Value Service Exposure Using Only More Physician-Driven Services
eTable 9. Average Adjusted CAHPS Scores by Low-Value Service Exposure, Based on Only More Physician-Driven Services
eAppendix 9. Additional Analysis: No Covariate Adjustment in CAHPS Model
eTable 10. Average Unadjusted CAHPS Scores by Low-Value Service Exposure
eAppendix 10. Description of all Code Files, With Website Address for GitLab Download
eTable 11. Files Related With Identifying Low-Value Services
eTable 12. Files Related With Identifying Primary Care Providers
eTable 13. Files Related With Applying Further Denominator Exclusions
eTable 14. Files Related With Creating Sample Summary Statistics and Adding Additional Covariates
eTable 15. Files Related With Modeling and Analysis

This supplementary material has been provided by the authors to give readers additional information about their work.

© 2021 Sanghavi P et al. *JAMA Internal Medicine*.

eAppendix 1. Identifying Primary Care Providers
We identified Primary Care Providers (PCPs) using provider specialty codes 01 (general practice), 08 (family practice), 11 (internal medicine), and 38 (geriatric medicine).

© 2021 Sanghavi P et al. *JAMA Internal Medicine*.

eAppendix 2. Missingness of CAHPS Items

eTable 1 describes the percent of missing responses by CAHPS item and year. The items in the composite for interactions with personal doctor had responses to all four items for 83.2% of respondents, to zero items for 15.8% of respondents, and to 1-3 items for 1% of respondents. In other words, individuals who responded at all to one of the items responded to all four items in a high proportion of cases.

eTable 1. Percent of Missing Responses by CAHPS Item and Year

	Pooled	2010	2011	2012	2013	2014	2015
Total N	379043	32968	81585	74992	72940	59733	56825
Single Items							
Healthcare rating	7%	13%	17%	3%	4%	3%	4%
Saw person came to see within 15 minutes of appt time	13%	13%	14%	12%	13%	12%	12%
Get appt for routine care as soon as needed	16%	16%	17%	16%	16%	16%	15%
Get care for illness as soon as needed	64%	65%	67%	65%	62%	64%	62%
Personal doctor rating	16%	18%	18%	15%	15%	15%	16%
Items in interactions with personal doctor composite							
MD Explains things	16%	18%	18%	15%	15%	15%	16%
MD Listens carefully	16%	18%	18%	15%	15%	15%	16%
MD Shows respect	16%	18%	18%	15%	15%	15%	16%
MD Spends enough time	16%	18%	18%	15%	15%	15%	16%

© 2021 Sanghavi P et al. *JAMA Internal Medicine*.

eAppendix 3. Low-Value Service Exposure Analytical Sample Flowchart
The below flowchart (eFigure 1) describes the starting sample of Medicare beneficiaries and the process by which observations were dropped to get to the final analytical sample for creating the low-value service exposure measures.

eFigure 1. Low-Value Service Exposure Analytical Sample Flowchart

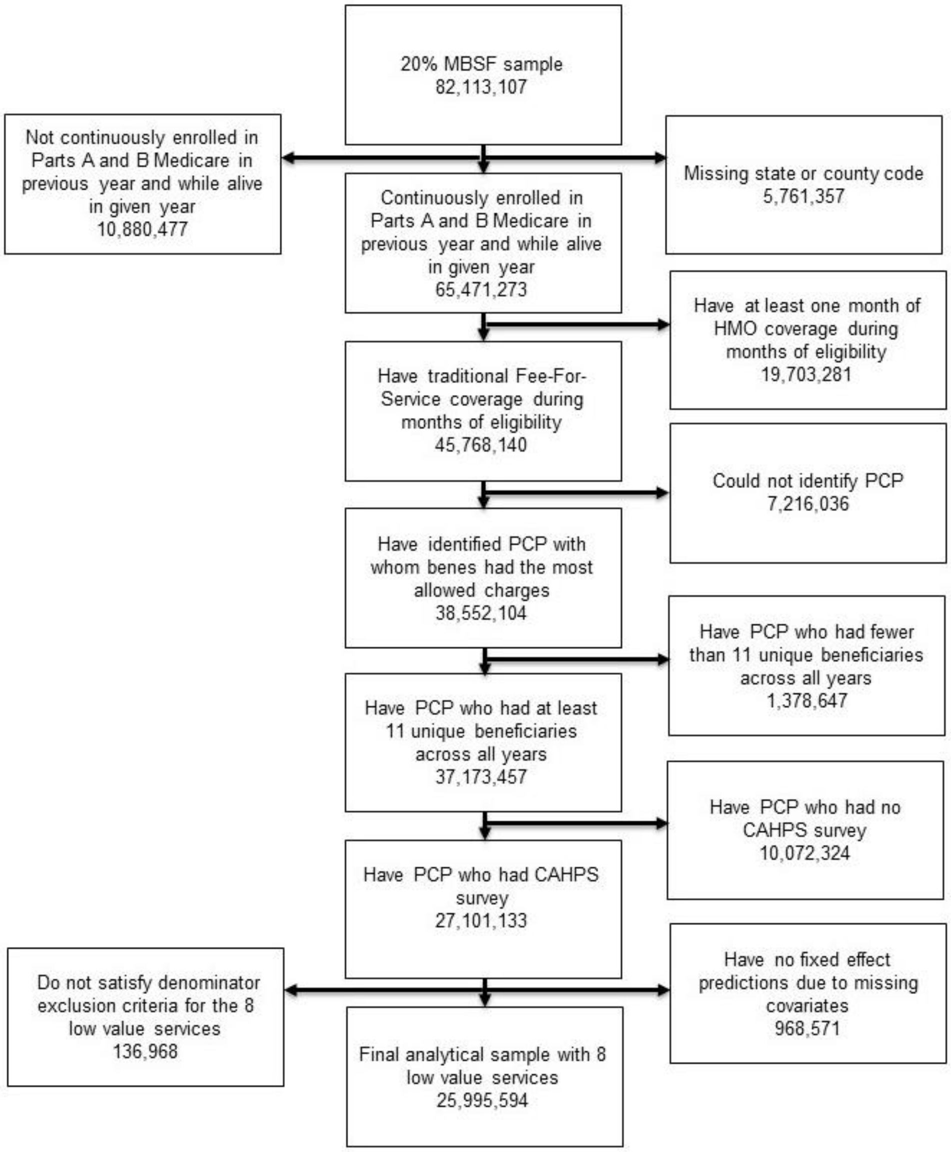

Notes

1. MBSF refers to Medicare Master Beneficiary Summary Files.
2. PCP refers to primary care physician.
3. CAHPS refers to Consumer Assessment of Healthcare Providers and Systems program.
4. Denominator count is based on Medicare Master Beneficiary Summary Files 2007-2014 at the bene-year level.

© 2021 Sanghavi P et al. *JAMA Internal Medicine*.

eAppendix 4. Comparison of Denominator Descriptions Used by Sanghavi et al. vs Schwartz et al [23]

We based the methodology for identifying low-value care on work by Schwartz et al. Services were considered low-value if they were identified as inappropriate within service-specific clinical scenarios by evidence-based lists like Choosing Wisely (https://www.choosingwisely.org/clinician-lists/) and recommendations from the U.S. Preventive

eTable 2. Comparison of Denominator Descriptions Used by Sanghavi et al. vs Schwartz et al **[23]**

	**Sanghavi et al.**	**Schwartz et al.**
**Prostate specific antigen (PSA) testing in older males**	Male patients 75 years and older with no history of prostate cancer	Men 75 and older
**Screening for carotid artery disease in asymptomatic adults**	Patients with no history of stroke or transient ischemic attack (TIA) prior to index year	All patients
**Cervical cancer screening for older females**	Female patients 65 years and older with no cervical cancer, dysplasia, diagnoses of other female genital cancers, abnormal Papanicolaou findings, or human papillomavirus positivity noted in index year's claims or in prior year's claims	Women over 65
**Parathyroid hormone (PTH) test for patients with stage 1-3 chronic kidney disease (CKD)**	Patients with CKD, with no hypercalcemia diagnosis noted in index year's claims	CKD patients not receiving dialysis
**Total or free T3 level testing for patients with hypothyroidism**	Patients with hypothyroidism diagnosis in index year's claims	Patients with hypothyroidism
**Back imaging for non-specific lower back pain**	Patients with no diagnoses for cancer, trauma, intravenous drug abuse, neurological impairment, endocarditis, septicemia, tuberculosis, osteomyelitis, fever, weight loss, loss of appetite, night sweats, anemia, radiculitis and myelopathy, and no back imaging after 6 weeks of first diagnosis of back pain, in index year's claims	All patients
**Head imaging for uncomplicated headache**	Patients with no diagnoses for thunderclap headache, epilepsy, giant cell arteritis, head trauma, convulsions, altered mental status, nervous system symptoms (e.g. hemiplegia), disturbances of skin sensation, speech problems, stroke/TIA, history of stroke, or cancer in index year's claims	All patients
**Spinal injection for lower back pain**	Patients with no diagnoses for radiculopathy in index year's claims, and no patients with spinal injections within 14 days after an inpatient stay	All patients

Services Task Forces (https://www.uspreventiveservicestaskforce.org/uspstf/recommendation-topics). Scenario criteria included factors like age, sex, service setting, timing relative to other

© 2021 Sanghavi P et al. *JAMA Internal Medicine*.

services, and diagnoses and procedures in the current and past year of claims, as well as indicators in the Chronic Conditions file.

Our analysis required not just identifying events of low-value care, but identifying denominator populations for whom service would be considered inappropriate. In the work by Schwarz et al., the interest was in providing rates of low-value care among Medicare beneficiaries broadly, and hence, denominators were defined broadly to include all patients or some subsets of patients (eTable 2). Importantly, these denominator definitions allowed individuals to be in the denominator for whom the service would not necessarily be considered low-value if received. For example, men with a history of prostate cancer would be included in the Schwartz et al. denominator, even though this is an exclusion for identifying low-value PSA testing. Therefore, for our purposes, we adapted the clinical scenarios for identifying low-value services to create denominator populations for whom the particular service would be considered low-value if received, as shown in eTable 2.

© 2021 Sanghavi P et al. *JAMA Internal Medicine*.

eAppendix 5. Low-Value Service Exposure Modeling Details

For computational feasibility, the implementation of the low-value service exposure composite model required two sub-steps. First, we modeled the fixed effects portion of the model, that is X_{ijst} in the model in the manuscript, separately for each low-value service and included linear predictors from these logistic models as covariates in the main model. Though the number of covariates here is not extraordinary, their combination with the large number of random effects made fitting the model a computationally intense task. Second, we separately fit the model shown in the main manuscript for each of 101 samples of about 1,000 physicians each, created by randomly sampling from three practice size-based strata. We included indicators for each of these samples as covariates when we modeled the associations between CAHPS measures and low-value service composites.

© 2021 Sanghavi P et al. *JAMA Internal Medicine*.

eAppendix 6. Average Characteristics of PCP Patient Panels in First and Fifth Low-Value Service Exposure Composite Quintiles

eTables 3 and 4 provide average characteristics of patient panels in the first and fifth quintiles of the low-value service exposure composites. eTable 3 provides averages of average PCP patient panels, i.e. each PCP patient panel gets equal weight; eTable 4 provides average characteristics across all patients, i.e. each PCP patient panel gets weighted according to the number of beneficiaries in it.

eTable 3. Average Characteristics of PCP Patient Panels in First and Fifth Low-Value Service Exposure Composite Quintiles, Unweighted

		LVS 1st quintile		LVS 5th quintile	
		Mean	SD	Mean	SD
Number of unique patients		35.2	25.6	38.8	28.4
Number of low-value service visits		392.9	232.5	432.2	268.3
Age		73.1	5.2	73.2	4.8
Female (%)		57.2	16.9	59.4	16.7
Race	White	82.8	23.8	85.2	20.6
	Asian	2.1	8.2	1.8	7.5
	Black	9.9	18.5	8.7	16.4
	Hispanic	1.7	6.2	1.7	6.2
Dual at least one month (%)		20.6	18.5	18.1	18.3
Total chronic conditions		6.3	1.1	6.7	1.2
Physician total charge		115948.5	79517.6	145639.5	107668.6
Total low-value service charge related Medicare Spending		3657841.4	2495547.4	4369269.4	3027564.5
HCC score		1.2	0.3	1.3	0.3
Percent college		19.0	8.5	19.5	8.4
Percent less than high school		33.5	11.2	32.2	10.8
Percent household median income		35225.9	9331.8	36111.8	9504.9
Percent in poverty		9.3	4.8	8.8	4.3
Percent live alone		27.5	4.9	27.2	4.4
**CAHPS Ratings**					
Overall Rating	Rating of health care	8.6	0.1	8.6	0.1
	Rating of primary physician	9.1	0.1	9.1	0.1
Access to Care	Timely Access to routine care	8.3	0.0	8.3	0.1
	Timely access to urgent care	8.8	0.0	8.8	0.0
Wait time		5.6	0.6	5.5	0.7
Interactions with primary physician	Composite score	9.0	0.1	9.0	0.1
	Clear communication	9.0	0.1	9.0	0.1
	Careful listening	9.1	0.1	9.1	0.1
	Respect	9.3	0.1	9.3	0.1
	Sufficient time	8.8	0.1	8.8	0.1

© 2021 Sanghavi P et al. *JAMA Internal Medicine*.

eTable 4. Average Characteristics of PCP Patient Panels in First and Fifth Low-Value Service Exposure Composite Quintiles, Weighted by Panel Size

	LVS 1st quintile		LVS 5th quintile	
	Mean	SD	Mean	SD
Number of low-value service visits per patient	5.6	2.2	5.7	2.2
Age	73.8	11.4	74.0	10.9
Female (%)	56.7	49.5	58.7	49.2
Race				
White	86.1	34.6	87.3	33.3
Asian	1.6	12.7	1.5	12.3
Black	8.4	27.7	7.6	26.4
Hispanic	1.2	10.8	1.3	11.2
Dual at least one month (%)	20.2	39.0	18.1	37.4
Total chronic conditions	6.1	3.3	6.5	3.3
Physician total charge	1715.9	1332.6	1957.3	1521.9
Total low-value service charge related Medicare Spending	47731.7	63356.0	52788.9	71513.3
HCC score	1.2	1.0	1.3	1.0
Percent college	18.6	11.8	19.5	12.0
Percent less than high school	33.6	14.9	32.2	14.9
Percent household median income	34931.2	13186.6	36127.6	13935.7
Percent in poverty	9.2	6.3	8.8	6.1
Percent live alone	27.4	8.1	27.0	7.9

© 2021 Sanghavi P et al. *JAMA Internal Medicine*.

eAppendix 7. Full Regression Tables

eTables 5-8 are regression tables for the main analysis described in the manuscript. eTable 5 provides the coefficients and p-values for covariates in the "fixed effects" portion of the low-value service exposure composite model (the first sub-step described above in section e5). Each column represents a regression for that particular low-value service. eTable 6 provides coefficients, p-values, and random effects variances for the main low-value service exposure composite model. This table shows one of the 101 samples described in section e5, but is fairly representative of other samples. eTables 7 and 8 are regression tables for the CAHPS items and composite.

eTable 5. Coefficients and *P* Values for "Fixed Effects" Sub-Step Regression by Low-Value Service (see eAppendix 5 to understand sub-step); log-odds ratios are shown

	psa	ctdasymp	cerv	pth	t3	backscan	head	spinj
(Intercept)	5.513 (0.08)	-11.34 (<2E16)	-1.79 (0.239)	-6.17 (<2E-16)	-0.259 (0.607)	-3.502 (<2E-16)	-1.531 (<0.001)	-4.837 (<2E-16)
age	-0.044 (0.559)	0.212 (<2E-16)	0.098 (0.014)	0.068 (<0.001)	-0.011 (0.365)	0.012 (0.095)	-0.064 (<2E-16)	-0.002 (0.876)
age2	< 0.001	-0.001 (<2E-16)	-0.001 (< 0.001)	-0.001 (<0.001)	< 0.001	< 0.001	< 0.001	< 0.001
female	NA	-0.099 (< 0.001)	NA	-0.037 (0.371)	0.178 (0.003)	0.106 (<0.001)	0.367 (<2E-16)	0.104 (0.022)
factor(race) black	-0.273 (0.023)	-0.216 (0.008)	0.142 (0.208)	0.104 (0.484)	0.367 (0.12)	0.172 (0.119)	0.174 (0.178)	-0.016 (0.938)
factor(race) hispanic	-0.122 (0.417)	-0.32 (0.005)	0.252 (0.092)	-0.071 (0.694)	-0.109 (0.687)	0.301 (0.022)	0.437 (0.002)	0.113 (0.632)
factor(race) other	-0.23 (0.081)	-0.126 (0.186)	-0.24 (0.077)	-0.05 (0.77)	-0.347 (0.207)	-0.048 (0.71)	0.043 (0.778)	0.211 (0.344)
factor(race) white	-0.184 (0.07)	0.019 (0.795)	0.123 (0.24)	-0.098 (0.487)	0.016 (0.942)	0.193 (0.062)	0.015 (0.903)	0.351 (0.065)
ccw_alzh_ demen	-0.304 (<0.001)	-0.436 (< 0.001)	-0.448 (< 0.001)	-0.412 (<0.001)	-0.001 (0.992)	-0.166 (0.002)	0.115 (0.047)	-0.375 (<0.001)
ccw_alzh	NA	NA	NA	NA	NA	NA	NA	NA
ccw_ami	-0.114 (0.014)	0.047 (0.226)	-0.167 (0.036)	-0.099 (0.066)	-0.008 (0.928)	-0.125 (0.013)	-0.128 (0.035)	-0.188 (0.022)
ccw_anemia	0.089 (0.001)	0.078 (< 0.001)	0.011 (0.665)	0.53 (<2E-16)	0.213 (<0.001)	0.018 (0.439)	0.131 (<0.001)	0.1 (0.006)
ccw_asthma	0.037 (0.354)	-0.073 (0.008)	0.141 (< 0.001)	-0.073 (0.08)	< 0.001	0.065 (0.03)	0.118 (0.001)	0.072 (0.094)
ccw_atrial_ fib	-0.039 (0.198)	-0.09 (0.001)	-0.022 (0.579)	-0.185 (<0.001)	0.012 (0.808)	0.058 (0.053)	0.093 (0.013)	-0.186 (<0.001)
ccw_cancer_. breast	-0.03 (0.917)	-0.102 (0.013)	0.242 (< 0.001)	-0.105 (0.127)	0.012 (0.852)	0.04 (0.346)	-0.095 (0.097)	-0.092 (0.187)
ccw_cancer_ colorectal	-0.043 (0.46)	-0.141 (0.007)	-0.048 (0.506)	0.02 (0.778)	-0.029 (0.775)	-0.096 (0.107)	-0.173 (0.03)	-0.167 (0.102)
ccw_cancer_ endometrial	-11.39 (0.972)	-0.185 (0.082)	0.442 (< 0.001)	0.025 (0.863)	-0.115 (0.468)	-0.097 (0.386)	-0.233 (0.113)	-0.128 (0.459)
ccw_cancer_ lung	-0.256 (0.008)	-0.185 (0.018)	-0.169 (0.135)	-0.391 (0.001)	-0.117 (0.413)	0.069 (0.431)	-0.024 (0.821)	-0.233 (0.114)
ccw_cancer_ prostate	NA	-0.118 (0.002)	-11.59 (0.955)	-0.223 (<0.001)	-0.09 (0.387)	0.05 (0.255)	-0.086 (0.204)	0.159 (0.026)
ccw_cataract	0.155 (<0.001)	0.055 (0.011)	0.209 (<2E-16)	-0.027 (0.45)	0.112 (0.01)	0.125 (<0.001)	0.066 (0.04)	0.036 (0.357)
ccw_chf	-0.233 (<0.001)	0.008 (0.738)	-0.152 (< 0.001)	0.091 (0.01)	-0.086 (0.065)	-0.019 (0.487)	0.004 (0.909)	-0.066 (0.122)
ccw_chronic kidney	-0.093 (0.002)	0.117 (< 0.001)	-0.037 (0.309)	0.662 (<2E-16)	-0.104 (0.024)	0.02 (0.479)	0.07 (0.04)	-0.025 (0.561)
ccw_copd	-0.008 (0.776)	0.1 (< 0.001)	-0.116 (< 0.001)	-0.127 (<0.001)	-0.076 (0.08)	0.027 (0.28)	0.133 (<0.001)	0.116 (0.002)
ccw_ depression	-0.128 (<0.001)	-0.094 (< 0.001)	-0.138 (< 0.001)	-0.305 (<2E-16)	-0.013 (0.73)	0.032 (0.176)	0.383 (<2E-16)	0.407 (<2E-16)
ccw_diabetes	-0.029 (0.26)	0.067 (0.001)	-0.21 (< 0.001)	0.223 (<0.001)	0.106 (0.006)	0.019 (0.397)	-0.005 (0.853)	-0.014 (0.699)
ccw_ glaucoma	0.115 (< 0.001)	0.063 (0.003)	0.122 (< 0.001)	0.011 (0.751)	-0.02 (0.619)	-0.025 (0.296)	0.062 (0.048)	0.045 (0.238)
ccw_hip_ fracture	-0.306 (< 0.001)	-0.273 (< 0.001)	-0.221 (0.007)	-0.249 (0.001)	-0.163 (0.088)	0.07 (0.211)	-0.159 (0.027)	-0.15 (0.103)
ccw_hyperl	0.398 (<2E-16)	0.463 (<2E-16)	0.197 (< 0.001)	0.434 (<0.001)	0.086 (0.109)	-0.014 (0.623)	-0.063 (0.086)	0.028 (0.541)
ccw_hyperp	0.591 (<2E-16)	0.053 (0.048)	0.876 (0.413)	-0.036 (0.409)	0.169 (0.019)	0.075 (0.022)	0.123 (0.008)	0.166 (0.002)
ccw_hypert	0.069 (0.092)	0.261 (<2E-16)	-0.172 (< 0.001)	0.789 (<0.001)	-0.148 (0.005)	-0.006 (0.83)	0.036 (0.381)	0.137 (0.005)
ccw_hypoth	0.023 (0.495)	-0.015 (0.488)	0.067 (0.006)	0.131 (<0.001)	-0.208 (<0.001)	0.022 (0.376)	0.119 (<0.001)	0.135 (<0.001)
ccw_ ischemicheart	-0.01 (0.737)	0.677 (<2E-16)	-0.052 (0.043)	-0.056 (0.132)	0.14 (0.001)	0.09 (<0.001)	0.288 (<2E-16)	0.168 (<0.001)
ccw_ osteoporosis	0.223 (< 0.001)	0.121 (< 0.001)	0.203 (<2E-16)	0.102 (0.008)	0.119 (0.003)	0.063 (0.016)	0.055 (0.097)	0.205 (<0.001)
ccw_ra_oa	0.046 (0.071)	0.032 (0.107)	0.145 (< 0.001)	-0.108 (0.001)	0.021 (0.589)	0.134 (<0.001)	0.3 (<2E-16)	1.556 (<2E-16)
ccw_stroke_ tia	-0.096 (0.002)	NA	-0.08 (0.032)	-0.133 (<0.001)	-0.103 (0.036)	0.039 (0.175)	0.291 (<2E-16)	-0.051 (0.26)
ccw_6up	-0.118 (0.005)	0.092 (0.003)	-0.012 (0.724)	-0.302 (<0.001)	-0.067 (0.284)	0.066 (0.049)	-0.013 (0.768)	0.005 (0.922)
hcc_t1	-0.211 (<2E-16)	0.029 (0.005)	-0.088 (<0.001)	0.124 (<2E-16)	0.006 (0.711)	-0.019 (0.11)	0.106 (<2E-16)	0.041 (0.013)
mcaid	-0.049 (0.347)	-0.122 (<0.001)	-0.544 (<2E-16)	-0.19 (<0.001)	-0.209 (<0.001)	-0.02 (0.54)	0.215 (<0.001)	-0.257 (<0.001)
factor(year) 2008	-0.174 (< 0.001)	0.009 (0.807)	-0.129 (0.001)	0.124 (0.093)	-0.001 (0.991)	-0.054 (0.163)	-0.122 (0.022)	0.001 (0.994)
factor(year) 2009	-0.174 (< 0.001)	-0.04 (0.271)	-0.093 (0.015)	0.161 (0.025)	0.097 (0.257)	-0.207 (<0.001)	-0.026 (0.621)	-0.022 (0.745)
factor(year) 2010	-0.272 (< 0.001)	0.011 (0.759)	-0.211 (<0.001)	0.249 (<0.001)	0.2 (0.017)	-0.11 (0.005)	-0.033 (0.519)	-0.021 (0.753)
factor(year) 2011	-0.325 (< 0.001)	-0.037 (0.31)	-0.274 (<0.001)	0.147 (0.034)	0.249 (0.002)	-0.179 (<0.001)	-0.225 (<0.001)	-0.001 (0.983)
factor(year) 2012	-0.559 (<2E-16)	-0.077 (0.035)	-0.435 (<2E-16)	0.122 (0.079)	0.353 (<0.001)	-0.207 (<0.001)	-0.169 (0.001)	0.007 (0.912)
factor(year) 2013	-0.632 (<2E-16)	-0.138 (< 0.001)	-0.689 (<2E-16)	0.143 (0.038)	0.232 (0.002)	-0.285 (<0.001)	-0.184 (0.001)	0.012 (0.85)
factor(year) 2014	-0.756 (<2E-16)	-0.145 (<0.001)	-0.87 (<2E-16)	0.163 (0.017)	0.214 (0.005)	-0.268 (<0.001)	-0.194 (<0.001)	0.002 (0.973)
zip5_hhinc_ mdn	< 0.001	< 0.001	< 0.001	< 0.001	< 0.001	< 0.001	< 0.001	< 0.001
zip5_in_ poverty	-0.002 (0.422)	-0.005 (0.024)	< 0.001	-0.007 (0.038)	-0.023 (<0.001)	0.001 (0.796)	0.001 (0.831)	-0.001 (0.744)
zip5_college	0.002 (0.174)	-0.005 (< 0.001)	0.009 (<0.001)	0.005 (0.019)	0.001 (0.72)	-0.003 (0.071)	-0.008 (<0.001)	0.004 (0.103)
HRR	Yes	Yes	Yes	Yes	Yes	Yes	Yes	Yes

Notes

© 2021 Sanghavi P et al. *JAMA Internal Medicine*.

1. Low value services are abbreviated as follows: psa refers to prostate specific antigen (PSA) testing in older males; ctdasymp refers to screening for carotid artery disease in asymptomatic adults; cerv refers to cervical cancer screening for older females; pth refers to parathyroid hormone (PTH) test for patients with stage 1-3 chronic kidney disease (CKD); t3 refers to total or free T3 level testing for patients with hypothyroidism; backscan refers to back imaging for non-specific lower back pain, head refers to head imaging for uncomplicated headache; spinj refers to spinal injection for lower back pain.

© 2021 Sanghavi P et al. *JAMA Internal Medicine*.

eTable 6. Coefficients and *P* Values for Low-Value Service Exposure Composite Model (this is one of the 101 samples described in eAppendix 5); log-odds ratios are shown

	Coef.	P-value
cerv	0.1855	0.075
ctdasym	0.0595	0.567
head	0.0467	0.693
psa	0.2497	0.013
pth	0.1388	0.202
spinj	0.0208	0.862
t3	0.1529	0.149
predictions	0.9948	0.000
predictions*cerv	0.0596	0.090
predictions*ctdasym	0.0147	0.669
predictions*head	0.0175	0.637
predictions*psa	0.1082	0.003
predictions*pth	0.0546	0.158
predictions*spinj	0.0111	0.763
predictions*t3	0.0562	0.142
Intercept	-0.2762	0.006
	Variance	
NPI-level random effects	0.053	
Patient nested within physician random effects	0.536	

Notes

1. Low value services are abbreviated as follows: psa refers to prostate specific antigen (PSA) testing in older males; ctdasymp refers to screening for carotid artery disease in asymptomatic adults; cerv refers to cervical cancer screening for older females; pth refers to parathyroid hormone (PTH) test for patients with stage 1-3 chronic kidney disease (CKD); t3 refers to total or free T3 level testing for patients with hypothyroidism; backscan refers to back imaging for non-specific lower back pain, head refers to head imaging for uncomplicated headache; spinj refers to spinal injection for lower back pain. Predictions are linear predictors of the “fixed effects” models shown in eTable 5.

© 2021 Sanghavi P et al. *JAMA Internal Medicine*.

eTable 7. Coefficients, Standard Errors, and *P* Values for CAHPS Linear Regressions for Non-Composite Items

CAHPS Items	Healthcare rating			Seen within 15 minutes			Routine care as soon as needed			Illness care as soon as needed			Personal doctor rating		
F-test for joint significance	F-statistic	p		F-statistic	p		F-statistic	p		F-statistic	p		F-statistic	p	
	1.510	0.137		37.210	<.0001		0.880	0.542		0.910	0.512		3.560	0.000	
**Parameter**	**Estimate**	**StdErr**	***Probt***	**Estimate**	**StdErr**	***Probt***	**Estimate**	**StdErr**	***Probt***	**Estimate**	**StdErr**	***Probt***	**Estimate**	**StdErr**	***Probt***
Decile 1	9.160	0.033	<.0001	6.540	0.085	<.0001	8.594	0.052	<.0001	8.933	0.076	<.0001	9.471	0.032	<.0001
Decile 2	9.153	0.033	<.0001	6.504	0.085	<.0001	8.560	0.053	<.0001	8.936	0.076	<.0001	9.446	0.032	<.0001
Decile 3	9.155	0.033	<.0001	6.526	0.085	<.0001	8.596	0.052	<.0001	8.934	0.076	<.0001	9.459	0.032	<.0001
Decile 4	9.152	0.033	<.0001	6.482	0.085	<.0001	8.572	0.052	<.0001	8.943	0.076	<.0001	9.482	0.032	<.0001
Decile 5	9.157	0.033	<.0001	6.466	0.085	<.0001	8.571	0.052	<.0001	8.953	0.076	<.0001	9.481	0.032	<.0001
Decile 6	9.150	0.033	<.0001	6.467	0.086	<.0001	8.570	0.052	<.0001	8.953	0.075	<.0001	9.489	0.032	<.0001
Decile 7	9.155	0.033	<.0001	6.384	0.085	<.0001	8.559	0.052	<.0001	8.956	0.075	<.0001	9.495	0.032	<.0001
Decile 8	9.155	0.033	<.0001	6.371	0.085	<.0001	8.590	0.052	<.0001	8.948	0.076	<.0001	9.497	0.032	<.0001
Decile 9	9.135	0.033	<.0001	6.263	0.085	<.0001	8.582	0.052	<.0001	8.991	0.075	<.0001	9.488	0.032	<.0001
Decile 10	9.125	0.033	<.0001	6.092	0.085	<.0001	8.581	0.052	<.0001	8.963	0.076	<.0001	9.470	0.032	<.0001
age_lt65	-0.171	0.015	<.0001	-0.248	0.028	<.0001	-0.064	0.021	0.003	-0.282	0.028	<.0001	-0.009	0.014	0.492
age_7074	0.062	0.008	<.0001	0.017	0.018	0.346	0.022	0.013	0.095	0.050	0.019	0.008	0.013	0.007	0.072
age_7579	0.114	0.009	<.0001	-0.001	0.018	0.955	-0.002	0.014	0.892	0.073	0.019	0.000	0.046	0.008	<.0001
age_8084	0.161	0.009	<.0001	0.026	0.020	0.182	-0.010	0.015	0.495	0.093	0.020	<.0001	0.068	0.008	<.0001
age_ge85	0.153	0.010	<.0001	0.056	0.020	0.006	0.008	0.016	0.593	0.171	0.021	<.0001	0.041	0.009	<.0001
less_8th	-0.174	0.019	<.0001	-0.217	0.035	<.0001	-0.249	0.028	<.0001	-0.188	0.035	<.0001	0.002	0.016	0.910
some_hs	-0.059	0.013	<.0001	-0.019	0.025	0.444	-0.147	0.020	<.0001	-0.090	0.025	0.000	0.034	0.011	0.003
somecoll	-0.064	0.007	<.0001	-0.035	0.015	0.021	0.022	0.012	0.062	-0.020	0.015	0.194	-0.071	0.007	<.0001
collgrad	-0.086	0.009	<.0001	0.011	0.019	0.578	0.021	0.015	0.154	0.071	0.020	0.000	-0.137	0.008	<.0001
collmore	-0.139	0.008	<.0001	0.006	0.017	0.737	0.025	0.013	0.052	0.028	0.018	0.114	-0.172	0.007	<.0001
edu_mis	-0.285	0.015	<.0001	-0.252	0.028	<.0001	-0.238	0.022	<.0001	-0.273	0.030	<.0001	-0.150	0.013	<.0001
medicaid	-0.121	0.014	<.0001	0.062	0.025	0.014	-0.148	0.020	<.0001	-0.199	0.025	<.0001	-0.015	0.013	0.227
ghs_vygd	-0.024	0.010	0.020	-0.215	0.024	<.0001	0.078	0.018	<.0001	0.170	0.026	<.0001	-0.041	0.009	<.0001
ghs_good	-0.237	0.011	<.0001	-0.598	0.024	<.0001	0.003	0.018	0.883	0.044	0.026	0.093	-0.157	0.009	<.0001
ghs_fair	-0.453	0.012	<.0001	-0.925	0.026	<.0001	-0.060	0.020	0.003	-0.085	0.028	0.002	-0.259	0.011	<.0001
ghs_poor	-0.614	0.019	<.0001	-1.074	0.034	<.0001	0.030	0.027	0.262	-0.082	0.034	0.016	-0.296	0.017	<.0001
mhs_vygd	-0.264	0.007	<.0001	-0.146	0.015	<.0001	-0.254	0.011	<.0001	-0.181	0.015	<.0001	-0.214	0.006	<.0001
mhs_good	-0.474	0.008	<.0001	-0.256	0.017	<.0001	-0.448	0.013	<.0001	-0.377	0.017	<.0001	-0.367	0.007	<.0001
mhs_fair	-0.668	0.013	<.0001	-0.374	0.024	<.0001	-0.547	0.019	<.0001	-0.458	0.024	<.0001	-0.513	0.012	<.0001
mhs_poor	-0.897	0.028	<.0001	-0.490	0.046	<.0001	-0.521	0.035	<.0001	-0.568	0.043	<.0001	-0.639	0.026	<.0001
Decile as numerical variable															
Decile	-0.003	0.001	0.006	-0.041	0.002	<.0001	-0.0002	0.002	0.915	0.005	0.002	0.022	0.003	0.001	0.001

© 2021 Sanghavi P et al. *JAMA Internal Medicine*.

eTable 8. Coefficients, Standard Errors, and *P* Values for CAHPS Linear Regressions for Interactions With Personal Doctor Composite

**CAHPS Items**	**MD Explains things**			**MD Listens carefully**			**MD Shows respect**			**MD Spends enough time**			**Interactions with personal doctor composite**		
**F-test for joint significance**	**F-statistic**	**p**		**F-statistic**	**p**		**F-statistic**	**p**		**F-statistic**	**p**		**F-statistic**	**p**	
	2.930	0.002		2.540	0.007		2.010	0.034		4.740	<.0001		3.550	0.000	
**Parameter**	**Estimate**	**StdErr**	***Probt***	**Estimate**	**StdErr**	***Probt***	**Estimate**	**StdErr**	***Probt***	**Estimate**	**StdErr**	***Probt***	**Estimate**	**StdErr**	**Probt**
Decile 1	9.523	0.041	*<.0001*	9.558	0.041	*<.0001*	9.651	0.036	*<.0001*	9.486	0.046	*<.0001*	9.545	0.035	<.0001
Decile 2	9.483	0.041	*<.0001*	9.528	0.041	*<.0001*	9.639	0.036	*<.0001*	9.445	0.046	*<.0001*	9.513	0.035	<.0001
Decile 3	9.497	0.041	*<.0001*	9.534	0.041	*<.0001*	9.639	0.036	*<.0001*	9.455	0.046	*<.0001*	9.522	0.035	<.0001
Decile 4	9.523	0.041	*<.0001*	9.553	0.041	*<.0001*	9.669	0.036	*<.0001*	9.474	0.046	*<.0001*	9.545	0.035	<.0001
Decile 5	9.510	0.041	*<.0001*	9.534	0.041	*<.0001*	9.650	0.036	*<.0001*	9.442	0.046	*<.0001*	9.523	0.035	<.0001
Decile 6	9.522	0.041	*<.0001*	9.543	0.041	*<.0001*	9.657	0.036	*<.0001*	9.458	0.045	*<.0001*	9.535	0.034	<.0001
Decile 7	9.539	0.041	*<.0001*	9.560	0.041	*<.0001*	9.660	0.036	*<.0001*	9.468	0.046	*<.0001*	9.547	0.035	<.0001
Decile 8	9.514	0.041	*<.0001*	9.547	0.041	*<.0001*	9.667	0.036	*<.0001*	9.457	0.046	*<.0001*	9.537	0.034	<.0001
Decile 9	9.486	0.041	*<.0001*	9.524	0.041	*<.0001*	9.640	0.036	*<.0001*	9.420	0.046	*<.0001*	9.507	0.035	<.0001
Decile 10	9.490	0.041	*<.0001*	9.504	0.041	*<.0001*	9.624	0.036	*<.0001*	9.391	0.046	*<.0001*	9.493	0.035	<.0001
age_lt65	0.046	0.017	*0.007*	0.002	0.017	*0.906*	-0.051	0.016	*0.002*	-0.030	0.019	*0.106*	-0.009	0.015	0.529
age_7074	-0.063	0.010	*<.0001*	-0.024	0.010	*0.016*	-0.031	0.009	*0.001*	-0.048	0.011	*<.0001*	-0.042	0.008	<.0001
age_7579	-0.111	0.010	*<.0001*	-0.018	0.010	*0.083*	-0.036	0.009	*<.0001*	-0.070	0.012	*<.0001*	-0.061	0.009	<.0001
age_8084	-0.157	0.011	*<.0001*	-0.016	0.011	*0.141*	-0.037	0.010	*0.000*	-0.107	0.013	*<.0001*	-0.081	0.010	<.0001
age_ge85	-0.244	0.012	*<.0001*	-0.040	0.012	*0.001*	-0.041	0.011	*<.0001*	-0.130	0.013	*<.0001*	-0.118	0.010	<.0001
less_8th	-0.089	0.022	*<.0001*	-0.022	0.020	*0.269*	0.029	0.019	*0.125*	0.023	0.022	*0.304*	-0.018	0.017	0.294
some_hs	-0.004	0.015	*0.799*	0.021	0.014	*0.139*	-0.002	0.013	*0.866*	0.047	0.016	*0.003*	0.013	0.012	0.286
somecoll	0.002	0.009	*0.778*	-0.076	0.009	*<.0001*	-0.039	0.008	*<.0001*	-0.085	0.010	*<.0001*	-0.049	0.007	<.0001
collgrad	0.000	0.011	*0.996*	-0.091	0.011	*<.0001*	-0.027	0.010	*0.008*	-0.110	0.013	*<.0001*	-0.056	0.010	<.0001
collmore	0.011	0.010	*0.274*	-0.103	0.010	*<.0001*	-0.004	0.009	*0.622*	-0.124	0.011	*<.0001*	-0.054	0.008	<.0001
edu_mis	-0.163	0.017	*<.0001*	-0.160	0.017	*<.0001*	-0.126	0.015	*<.0001*	-0.182	0.019	*<.0001*	-0.164	0.015	<.0001
medicaid	-0.069	0.016	*<.0001*	-0.022	0.015	*0.147*	-0.056	0.015	*0.000*	-0.066	0.017	*<.0001*	-0.055	0.014	<.0001
ghs_vygd	-0.006	0.012	*0.628*	-0.037	0.012	*0.001*	-0.006	0.010	*0.573*	-0.065	0.013	*<.0001*	-0.019	0.010	0.055
ghs_good	-0.153	0.012	*<.0001*	-0.175	0.012	*<.0001*	-0.109	0.011	*<.0001*	-0.235	0.014	*<.0001*	-0.158	0.010	<.0001
ghs_fair	-0.292	0.014	*<.0001*	-0.294	0.014	*<.0001*	-0.212	0.012	*<.0001*	-0.379	0.015	*<.0001*	-0.285	0.012	<.0001
ghs_poor	-0.361	0.021	*<.0001*	-0.355	0.021	*<.0001*	-0.291	0.020	*<.0001*	-0.430	0.023	*<.0001*	-0.352	0.019	<.0001
mhs_vygd	-0.259	0.008	*<.0001*	-0.257	0.008	*<.0001*	-0.211	0.007	*<.0001*	-0.310	0.009	*<.0001*	-0.259	0.007	<.0001
mhs_good	-0.483	0.010	*<.0001*	-0.433	0.010	*<.0001*	-0.384	0.009	*<.0001*	-0.501	0.011	*<.0001*	-0.451	0.008	<.0001
mhs_fair	-0.666	0.016	*<.0001*	-0.630	0.016	*<.0001*	-0.554	0.015	*<.0001*	-0.694	0.017	*<.0001*	-0.638	0.014	<.0001
mhs_poor	-0.815	0.033	*<.0001*	-0.769	0.033	*<.0001*	-0.663	0.031	*<.0001*	-0.858	0.036	*<.0001*	-0.779	0.029	<.0001
Decile as numerical variable															
Decile	-0.001	0.001	0.502	-0.003	0.001	0.031	-0.001	0.001	0.527	-0.006	0.001	<.0001	-0.003	0.001	0.017

© 2021 Sanghavi P et al. *JAMA Internal Medicine*.

eAppendix 8. Additional Analysis: Estimating Low-Value Service Exposure Using Only More Physician-Driven Services

In this analysis, we limited our low-value care services to only those that are less sensitive to patient preferences[4], that is carotid artery disease screening in asymptomatic patients, PTH testing for CKD, and T3 testing for hypothyroidism, and re-created the exposure composite measures. The results of this analysis are shown in eTable 9 and are consistent with our main findings.

eTable 9. Average Adjusted[1] CAHPS[2] Scores by Low-Value Service Exposure, Based on Only More Physician-Driven Services[3]

More low-value care exposure

	A. Your Health Care in the Last 6 Months				B. Your Personal Doctor	
Low-value service exposure	Overall healthcare	Wait time	Timely access to non-urgent care	Timely access to urgent care	Overall personal doctor	Interactions with personal doctor composite
**Deciles of low-value service exposure, specified as categorical variable[4]**						
1	9.185	6.477	8.620	8.964	9.498	9.564
2	9.168	6.520	8.586	8.928	9.476	9.540
3	9.166	6.549	8.591	8.958	9.477	9.545
4	9.177	6.534	8.581	8.952	9.484	9.552
5	9.147	6.497	8.560	8.967	9.472	9.535
6	9.146	6.461	8.557	8.961	9.485	9.542
7	9.133	6.437	8.566	8.927	9.478	9.534
8	9.149	6.346	8.560	8.950	9.484	9.532
9	9.127	6.274	8.576	8.952	9.479	9.537
10	9.104	6.036	8.581	8.961	9.457	9.497
F-statistic (p-value)	7.60 (*<.0001*)	48.21 (*<.0001*)	1.83 (*0.058*)	0.57 (*0.826*)	1.55 (*0.123*)	3.25 (*0.001*)
**Deciles of low-value service exposure, specified as continuous variable[5]**						
Low-value service exposure	-0.007 (*<.0001*)	-0.043 (*<.0001*)	-0.004 (*0.014*)	0.000 (*0.953*)	-0.002 (*0.034*)	-0.005 (*<.0001*)

Notes

1. Each CAHPS outcome was separately modeled with a linear regression that adjusted for age, Medicaid-Medicare dual status, highest level of education completed, overall health rating, and overall mental or emotional health rating, and included physician clustered standard errors.
2. CAHPS refers to the Medicare Fee-for-Service Consumer Assessment of Healthcare Providers & Systems.
3. The low-value services exposure composite is based on only these services, carotid artery disease screening in asymptomatic patients, PTH testing for CKD, and T3 testing for hypothyroidism, which are considered less sensitive to patient preferences [10].
4. Deciles of low-value service exposure were specified as categorical variables and the intercept was dropped to allow direct interpretation of decile coefficients as average adjusted CAHPS scores (rather than as comparisons to a reference category). F-tests were conducted to test the joint significance of the decile coefficients. As an example of interpretation, PCP patient panels in the fifth decile of low-value care exposure rated their overall healthcare 9.147 out of 10 on average, controlling for age, dual status, education, and overall health and mental or emotional health rating.
5. Deciles of low-value service exposure were specified as a continuous variable (integers 1 to 10). As an example of interpretation, PCP patient panels in one higher decile of low-value care exposure rated

© 2021 Sanghavi P et al. *JAMA Internal Medicine*.

their overall healthcare 0.007 points lower (on a 10 point scale) on average, controlling for age, dual status, education, and overall health and mental or emotional health rating.

© 2021 Sanghavi P et al. *JAMA Internal Medicine*.

eAppendix 9. Additional Analysis: No Covariate Adjustment in CAHPS Model

In this analysis, we removed all adjustments in our CAHPS models, and found no change to the implications of our findings.

eTable 10. Average Unadjusted[1] CAHPS[2] Scores by Low-Value Service Exposure

More low-value care exposure ↓

	A. Your Health Care in the Last 6 Months				B. Your Personal Doctor	
Low-value service exposure	Overall healthcare	Wait time	Timely access to non-urgent care	Timely access to urgent care	Overall personal doctor	Interactions with personal doctor composite
**Deciles of low-value service exposure, specified as categorical variable[2]**						
1	8.638	5.822	8.315	8.714	9.062	9.028
2	8.629	5.783	8.283	8.713	9.036	8.997
3	8.627	5.803	8.315	8.709	9.046	9.000
4	8.622	5.755	8.291	8.720	9.068	9.023
5	8.629	5.740	8.291	8.732	9.068	9.003
6	8.628	5.746	8.294	8.740	9.078	9.016
7	8.637	5.665	8.286	8.746	9.086	9.031
8	8.637	5.656	8.319	8.734	9.087	9.022
9	8.618	5.549	8.311	8.781	9.078	8.994
10	8.611	5.377	8.311	8.756	9.063	8.980
F-statistic (p-value)	0.87 *(0.550)*	34.52 *(<.0001)*	0.95 *(0.480)*	1.47 *(0.154)*	3.55 *(0.000)*	2.96 *(0.002)*
**Deciles of low-value service exposure, specified as continuous variable[3]**						
Low-value service exposure	-0.001 *(0.178)*	-0.040 *(<.0001)*	0.001 *(0.519)*	0.006 *(0.002)*	0.003 *(0.000)*	-0.002 *(0.099)*

Notes

1. Each CAHPS outcome was separately modeled with a linear regression and no other covariates, and included physician clustered standard errors.
2. CAHPS refers to the Medicare Fee-for-Service Consumer Assessment of Healthcare Providers & Systems.
3. Deciles of low-value service exposure were specified as categorical variables and the intercept was dropped to allow direct interpretation of decile coefficients as average adjusted CAHPS scores (rather than as comparisons to a reference category). F-tests were conducted to test the joint significance of the decile coefficients. As an example of interpretation, PCP patient panels in the fifth decile of low-value care exposure rated their overall healthcare 8.629 out of 10 on average.
4. Deciles of low-value service exposure were specified as a continuous variable (integers 1 to 10). As an example of interpretation, PCP patient panels in one higher decile of low-value care exposure rated their overall healthcare 0.001 points lower (on a 10 point scale) on average.

© 2021 Sanghavi P et al. *JAMA Internal Medicine*.

eAppendix 10. Description of all Code Files, With Website Address for GitLab Download

These notes briefly highlight the purposes of the code files that are in the tables in each section. All code files listed below are shareable per our Data Use Agreement (DUA) with the Centers for Medicare and Medicaid Services (CMS) and are publicly posted on our GitHub page: https://github.com/sanghavi-lab/low_value_services

Software

We used SAS 9.4, Stata/MP 15.0, and R Version 3.5.1 for this analysis.

Identifying Low-Value Services

All programs were made to be run on the 2006-2014 Medicare data, formatted like the data housed at the National Bureau of Economic Research (NBER). These programs search for low-value services occurring between 2007 and 2014. Note that searching for low-value services in a given year (the "index" year) also requires using data in a "lookback" year, the prior year. We first created Medicare file extracts containing variables needed for low-value service screening, then searched for low-value services, created beneficiary-level covariates for analysis, and combined outputs to create a beneficiary-year-level dataset with beneficiaries' low-value services and covariates. For each measure and each year, the program produced two sets of low-value services. One set has low-value services identified using a "specific" detection criteria and the other has low-value services identified using a "sensitive" detection criteria.

eTable 11. Files Related With Identifying Low-Value Services

Program name	Input files (File source)	Output files
Mcarextracts.sas	Beneficiary summary files for the index year and prior year<br>Carrier (line and claim), outpatient (line and claim), and MedPAR claims files for the index year and prior year<br>BETOS to HCPCS crosswalk for the index year and prior year	ourbenes\`year'_20.sas7bdat<br>car\`year'_20.sas7bdat<br>otpt\`year'_20.sas7bdat<br>medpar\`year'_20.sas7bdat
flags.sas	car\`year'_20.sas7bdat<br>otpt\`year'_20.sas7bdat<br>medpar\`year'_20.sas7bdat	fl_'measure_number'_\`year'_\`sensitivity_level'.sas7bdat
covars.sas	ourbenes\`year'_20.sas7bdat<br>car\`year'_20.sas7bdat<br>otpt\`year'_20.sas7bdat<br>medpar\`year'_20.sas7bdat	ourbenescovars.sas7bdat
flags2.sas	fl_'measure_number'_\`year'_\`sensitivity_level'.sas7bdat<br>ourbenescovars.sas7bdat	yranalysis_20.sas7bdat

Note: \`Year' ranges from 2007-2014. 'Measure_number' ranges from 1-31. \`Sensitivity_level' takes on values of "sensitive" and "specific".

Identify Primary Care Providers

We identified for each beneficiary his/her primary care provider as the provider (NPI) with whom the beneficiary had the most allowed charges on primary care claims within each year. To be

© 2021 Sanghavi P et al. *JAMA Internal Medicine*.

conservative with our analysis, we constructed our sample based on low-value services identified using the "specific" detection criteria. We limited our sample by only including NPIs with at least 11 patients in years 2007-2014, as per our DUA with CMS.

eTable 12. Files Related With Identifying Primary Care Providers

Program name	Input files (File source)	Output files
AssignNPI.sas	Carrier (line and claim) files	claims_npi_20.sas7bdat
MergeNPI.sas	claims_npi_20.sas7bdat<br>yranalysis_20.sas7bdat	bene_pcp_final.sas7bdat

Apply Further Denominator Exclusions

We applied finer exclusion criteria to the low-value service denominators following the denominator definitions in Table 1 of the manuscript. From the carrier, outpatient, and MedPAR claims we identified relevant claims for patients who should be excluded from each denominator for head imaging, back imaging, PAP test, PTH test, and spinal injection. We also used chronic condition flags to exclude patients with history of prostate cancer from PSA test, stroke or transient ischemic attack (TIA) from carotid artery screening.

eTable 13. Files Related With Applying Further Denominator Exclusions

Program name	Input files (File source)	Output files
Denomexclusion.sas	car`year'_20.sas7bdat<br>otpt`year'_20.sas7bdat<br>medpar`year'_20.sas7bdat<br>bsfcc`year'.sas7bdat	excervcar_`year'.sas7bdat<br>excncrpthcar_`year'.sas7bdat<br>expthcar_`year'.sas7bdat<br>exrhinoctcar_`year'.sas7bdat<br>exbackscancar_`year'.sas7bdat<br>excervotpt_`year'.sas7bdat<br>excncrpthotpt_`year'.sas7bdat<br>expthotpt_`year'.sas7bdat<br>exrhinoctotpt_`year'.sas7bdat<br>exbackscanotpt_`year'.sas7bdat<br>exrhcathmedpar_`year'.sas7bdat<br>cerv_ex.sas7bdat<br>hica.sas7bdat<br>stroke.sas7bdat
Denomexclusionflag.sas	car`year'_20.sas7bdat<br>otpt`year'_20.sas7bdat	backscan_ex.sas7bdat<br>head_ex.sas7bdat<br>spinj_ex.sas7bdat
Denommerge.sas	bene_pcp_final.sas7bdat<br>cerv_ex.sas7bdat<br>hica.sas7bdat<br>stroke.sas7bdat<br>backscan_ex.sas7bdat<br>head_ex.sas7bdat<br>spinj_ex.sas7bdat	bene_pcp_final_ex.sas7bdat

Note: `Year' ranges from 2007-2014.

Create Sample Summary Statistics and Add Additional Covariates

© 2021 Sanghavi P et al. *JAMA Internal Medicine*.

We calculated rates of low-value service utilization as a percentage of qualifying patients who received the service and identified the top eight low-value services with the highest utilization rates. We merged our sample of low-value services with CAHPS survey data by NPI and dropped observations for NPIs who have no CAHPS reviews. Then we created NPI deciles based on per NPI count of beneficiaries and generated 101 random samples stratifying on NPI deciles. Finally, we merged in HCC scores, chronic condition indicators, and zip-level socioeconomic variables and created eight output datasets for the top eight low-value services with the highest utilization rates.

eTable 14. Files Related With Creating Sample Summary Statistics and Adding Additional Covariates

Program name	Input files (File source)	Output files
Exhibit1.sas	bene_pcp_final_ex.sas7bdat	lvsuse_meancount_bin.csv
Sample.sas	bene_pcp_final_ex.sas7bdat	lvs_npisamp.sas7bdat
Addvar.sas	lvs_npisamp.sas7bdat	lvscle_backscan.sas7bdat<br>lvscle_cerv.sas7bdat<br>lvscle_ctdasym.sas7bdat<br>lvscle_head.sas7bdat<br>lvscle_psa.sas7bdat<br>lvscle_pth.sas7bdat<br>lvscle_spinj.sas7bdat<br>lvscle_t3.sas7bdat

Modeling and Analysis

To make it computationally viable, we ran a model with all fixed effects only to predict the outcome variable of whether patients received a low value service. In R, we used glm() to generate fixed effects predictions separately for each of the eight low value services on the linear (log-odds) scale. The fixed effects included patient characteristics (age, sex, race, chronic conditions indicators, and dual status), geographical characteristics, including household income, percent poverty, percent population with college/less than high school education, and percent population who live alone, year, and HRR (hospital referral regions). Predictions for each low value service at the bene-year level were then used in an interaction with low value service type in a mixed model with NPI random effects (with beneficiaries nested). We use melogit in Stata to run the three-level mixed-effects logistic regressions. The NPI-level random effects were saved as the low-value service exposure composites.

Using these low-value service exposure composites, we ranked NPIs into quintiles and deciles, for different purposes. For Figure 1, we calculated rates of receipts of individual low-value services for PCP patient panels by quintiles of the low-value service exposure composites. We centered and rescaled CAHPS measures on a 0 to 10 scale and created CAHPS score composites for interactions with the personal doctor. We regressed CAHPS measures on low value service exposure composites adjusting for patient characteristics (age, dual status, education, health status) using PROC MIXED procedure in SAS.

© 2021 Sanghavi P et al. *JAMA Internal Medicine*.

eTable 15. Files Related With Modeling and Analysis

Program name	Input files (File source)	Output files
Fepredict.R	lvscle_backscan.sas7bdat<br>lvscle_cerv.sas7bdat<br>lvscle_ctdasym.sas7bdat<br>lvscle_head.sas7bdat<br>lvscle_psa.sas7bdat<br>lvscle_pth.sas7bdat<br>lvscle_spinj.sas7bdat<br>lvscle_t3.sas7bdat	lvscle_backscan_fe.sas7bdat<br>lvscle_cerv_fe.sas7bdat<br>lvscle_ctdasym_fe.sas7bdat<br>lvscle_head_fe.sas7bdat<br>lvscle_psa_fe.sas7bdat<br>lvscle_pth_fe.sas7bdat<br>lvscle_spinj_fe.sas7bdat<br>lvscle_t3_fe.sas7bdat
addfixedpred.sas	lvscle_backscan.sas7bdat<br>lvscle_cerv.sas7bdat<br>lvscle_ctdasym.sas7bdat<br>lvscle_head.sas7bdat<br>lvscle_psa.sas7bdat<br>lvscle_pth.sas7bdat<br>lvscle_spinj.sas7bdat<br>lvscle_t3.sas7bdat<br>lvscle_backscan_fe.sas7bdat<br>lvscle_cerv_fe.sas7bdat<br>lvscle_ctdasym_fe.sas7bdat<br>lvscle_head_fe.sas7bdat<br>lvscle_psa_fe.sas7bdat<br>lvscle_pth_fe.sas7bdat<br>lvscle_spinj_fe.sas7bdat<br>lvscle_t3_fe.sas7bdat	lvscle_8measures.sas7bdat<br>lvscle`sample'.dta
Randomeffects.do	lvscle`sample'.dta	lvscle_re`sample'.csv
Exhibit3.sas	lvscle_re`sample'.csv<br>lvscle_8measures.sas7bdat	exhibit3.csv
Exhibit5.sas	lvscle_8measures.sas7bdat<br>lvscle_re`sample'.csv	exhibit5.csv
Regression.sas	lvscle_re`sample'.csv	exhibit4.csv

Note: `Sample' ranges from 1-101.

© 2021 Sanghavi P et al. *JAMA Internal Medicine*.
